# Anticoagulants and fracture morphology have a significant influence on total blood loss after proximal femur fractures

**DOI:** 10.1007/s00068-022-02090-y

**Published:** 2022-09-12

**Authors:** Annabel Fenwick, Michael Pfann, Jakob Mayr, Iana Antonovska, Andreas Wiedl, Malte Feldmann, Stefan Nuber, Stefan Förch, Edgar Mayr

**Affiliations:** Trauma, Orthopedic, Plastic and Hand Surgery, University Hospital of Augsburg, Stenglinstrasse 2, 86156 Augsburg, Germany

**Keywords:** Blood loss, Proximal femur fracture, Anticoagulants, Mortality

## Abstract

**Introduction:**

Blood loss after proximal femoral fractures is an important risk factor for postoperative outcome and recovery. The purpose of our study was to investigate the total blood loss depending on fracture type and additional risks, such as anticoagulant use, to be able to recognize vulnerable patients depending on planned surgery and underlying comorbidities.

**Materials and methods:**

A retrospective single center study including 1478 patients treated operatively for a proximal femoral fracture between January 2016 and June 2020 at a level I trauma center. Patient data, surgical procedure, time to surgery, complications and mortality were assessed. Lab data including hemoglobin and transfusion rates were collected. The Mercuriali formula was implemented to calculate total blood loss. Linear regression was performed to identify influencing factors.

**Results:**

One thousand four hundred seventy-eight mainly female patients were included in the study (mean age: 79.8 years) comprising 667 femoral neck fractures, 704 pertrochanteric- and 107 subtrochanteric fractures. Nearly 50% of the cohort were on anticoagulants or anti- platelet therapy. At time of admission average hemoglobin was 12.1 g/l. Linear regression proved fracture morphology, age, BMI, in-house mortality and anticoagulant use to have crucial influence on postoperative blood loss. Femoral neck fractures had a blood loss of 1227.5 ml (SD 740.4 ml), pertrochanteric fractures lost 1,474.2 ml (SD 830 ml) and subtrochanteric femoral fractures lost 1902.2 ml (SD 1,058 ml).

**Conclusions:**

Hidden blood loss is underestimated. Anticoagulant use, fracture type, gender and BMI influence the total blood loss. Hemoglobin levels should be monitored closely. Within 48 h there was no increased mortality, so adequate time should be given to reduce anticoagulant levels and safely perform surgery.

## Introduction

Hip fractures are common and increasing [1, 2]. In Germany, the incidence of proximal femur fractures has risen by 24% from 2009 to 2019 [3]. Surgery performed depending on fracture morphology is total- or hemi arthroplasty or intramedullary nailing. Mortality rates are as high as 30% during the first postoperative year [4]. Hidden blood loss was first proposed by Sehat et al. [5] after a study in total knee arthroplasty. Data have shown that the total blood loss to be up to 100% (knee)/30% (hip) higher than expected for planned knee/hip arthroplasty [5–7]. Few studies have investigated blood loss after femoral fractures [8]. The fracture itself as well as subsequent surgery can lead to significant blood loss and anemia and thus prolong postoperative recovery [9, 10]. Former studies have shown that the amount of actual blood loss is much higher than observed intraoperatively [11, 12]. Severe blood loss results in anemia and hypovolemia and can worsen cardiac symptoms in patients suffering from cardiac co- morbidities or renal dysfunction [13, 14]. Geriatric patients who are increasingly admitted with these types of fractures are especially vulnerable to these blood loss-associated problems. A rising number of patients on anticoagulants pose a further risk of an even higher blood loss.

Few studies have investigated blood loss after proximal femoral fractures differentiating between fracture morphology and additional risk factors such as anticoagulants. We aimed to compare all fracture types and their surgical procedures for proximal femoral fractures and differentiate between patients with and without anticoagulants to try and define risk factors and to be able to recognize vulnerable patients depending on planned surgery and underlying comorbidities early.

## Materials and methods

For our single center study all patients treated operatively for a proximal femoral fracture between January 2016 and June 2020 at a level I trauma center were reviewed. Femoral neck, pertrochanteric and subtrochanteric fractures were included. Greater trochanteric fractures, periprosthetic fractures as well as transfers for revision surgery and polytrauma patients were excluded. Patients without pre- or postoperative labs, concomitant fractures and patients undergoing further surgical procedures during the first 6 days after admission for proximal femoral fracture were excluded to avoid confounding factors.

The study conducted was approved by the local Ethics Committee and fulfils the standards of the declaration of Helsinki (20-2155-101).

The charts were reviewed for age, gender, BMI, fracture morphology, medication, revisions, labs and blood transfusions. If patients were admitted again for the contralateral side during the reviewed period they were included again as a separate case.

**Fig. 1:**
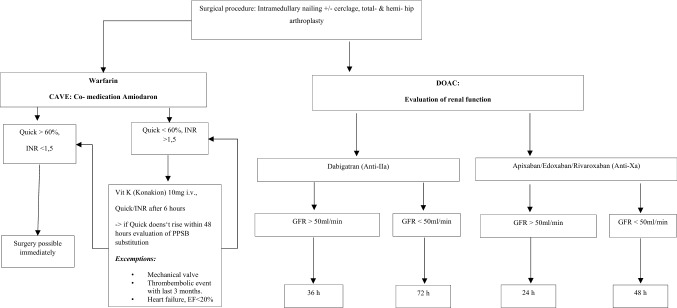
Standard protocol for proximal femoral fractures and anticoagulants

According to pre-operative mobility and comorbidities as well as fracture morphology total or hemi arthroplasty (cemented or uncemented) was performed for femoral neck fractures and intramedullary nailing PFNa (± cerclage) for pertrochanteric fractures. All subtrochanteric fractures were addressed by open reduction, cerclage and intramedullary nailing in side-positioning. Patients without anticoagulants or on anti-platelet medication were treated within 24 h. Last administration of anticoagulants was recorded. Patients on anticoagulants were operated on within 24–72 h depending on last dose, renal function, and type of anticoagulant according to our in-house standard protocol (Fig. 1). Postoperatively venous thromboembolism prophylaxis was given from day one with Enoxaparin 40 mg subcutaneously and anticoagulants were substituted with Innohep according to patient weight. Mobilization was initiated from day one after surgery for all patients.

### Mercuriali formula

Preoperative labs were taken as well as postoperative labs on day one and day five including hemoglobin and hematocrit. Transfusions were performed if the hemoglobin level dropped below 7.0 g/l and/or patients presented symptoms of anemia especially in the presence of cardiac co-morbidities. The blood loss was calculated using the Mercuriali formula [15], which is based on pre- and postoperative hematocrit and the number of transfused RBCs (Red blood cell, 300 ml) as well as patients’ blood volume. This is calculated by the Nadler formula [16], which is a specific calculation according to gender and height.

$${\text{Women}}:\;{\text{BV}}\left( {\text{l}} \right) = {\text{Height }}\left( {\text{m}} \right)^{{3}} - 0.{3561} + {\text{weight }}\left( {{\text{kg}}} \right) - 0.0{33}0{8} + 0.{1833}$$

$${\text{Men}}:\;{\text{BV}}\left( {\text{l}} \right) = {\text{Height}}\left( {\text{m}} \right)^{{3}} - 0{3669} + {\text{weight}}\left( {{\text{kg}}} \right) - 0.0{3219} + 0.{6}0{41}$$

$${\text{Estimated blood loss}}:\;{\text{BV}} \times \left( {{\text{Hct}}_{{{\text{preop}}}} {-}{\text{Hct}}_{{\text{day 5 postoperative}}} } \right) + {\text{ml of transfused RBC}}$$

### Statistical analysis

Statistical analysis was carried out with IBM SPSS Statistics (version 27; IBM Deutschland Ltd., Ehningen, Germany). Normal distribution of all data was verified with Shapiro Wilk Test. The student’s *t* test, Chi square, ANOVA variance and binary logistic regression were used to determine differences and influencing factors regarding complications and mortality. For data without normal distribution the Wilcoxon Rank Test was used. 95% confidence intervals and standard deviations were calculated. The significance level was set at 5% (*α* = 0.05).

## Results

One thousand four hundred seventy-eight patients were included in the investigation. 68.9%: were female and 31.1%: male with an average age of 79.8 years (range: 18–103; SD 12). The mean BMI was 24.38 kg/m^2^ (range: 11.7–66 kg/m^2^). The cohort consisted of 667 femoral neck fractures, 704 pertrochanteric- and 107 subtrochanteric fractures. In 335 cases a total hip endoprosthesis was implanted. 332 patients received a hemiarthroplasty. Intramedullary nailing was performed in 811 cases. Between the fracture types there were no statistical differences for gender or BMI distribution. 62.9% of all patients could be treated within 24 h and another 25.8% met the 48-h time limit. The average waiting time for surgery was 25.9 h (SD 20.2 h) after hospital admission. The total complication rate was 21.9% (surgical site infection, urinary tract infection, pneumonia, pulmonary embolism, thrombosis, dislocation, fracture), Table 1.

**Table 1:** Time to surgery, complications and blood loss according to anticoagulant therapy

	No anticoagulants	Antiplatelet therapy	Warfarin	DOAC
Number of patients (with subgroups)	741	464	93	180
		ASS *N* = 420		Rivaroxaban *N* = 61
		Clopidogrel		Edoxaban *N* = 33
		*N* = 31		Apixaban *N* = 82
		ASS + Clopidogrel		Dabigatran *N* = 4
		*N* = 13		
Time to surgery (in hours)	22.1	23.5	42.9	39.3
Complications with treatment required (in%)				
Pneumonia	47 (6.3)	26 (5.6)	9 (9.7)	14 (7.7)
Urinary tract inf	76 (10.3)	51 (10.9)	20 (21.5)	14 (7.7)
Wound infection	22 (2.9)	10 (2.2)	2 (2.1)	10 (5.5)
Hematoma	18 (2.4)	8 (1.7)	9 (9.7)	12 (6.7)
Mortality	20 (2.7)	11 (2.4)	3 (3.2)	10 (5.5)
Blood loss in ml	1315.5	1428.8	1620.5	1509.4
Number of patients receiving RBC transfusion	223 (30.0)	147 (31.17)	36 (38.7)	141 (37.2)

50% of the cohort had no rheological therapy. 18.4% (*N* = 273) of the cohort were on anticoagulants at admission. Of these patients 180 were on DOACs, 93 on Warfarin. A further 464 patients were receiving anti-platelet therapy (29.9%).

On average patients on anticoagulants had a delay to surgery of 41.37 h vs 22.1 h for patients not on anticoagulants and 23.5 h for patients with anti-platelet therapy (*p* < 0.000). The presence of anticoagulant therapy displayed a significant correlation with the occurrence of complications (*p* < 0.013) including pneumonia and postoperative hematoma in need of revision surgery. Hematoma, especially, occurred more often in patients on DOACs or Warfarin. The overall mortality rate in this cohort was 2.98% (*N* = 44). The mortality rate for patients on Warfarin (3.2%) and DOACs (5.5%) was slightly higher but not statistically significant (*p* < 0.219); Table 1. The mortality rate did not differ for patients operated on within 24 or 48 h (2.3 vs. 2.9%). After 48 h there was an increase in mortality rate to 6.2% (*p* < 0.085).

**Fig. 2:**
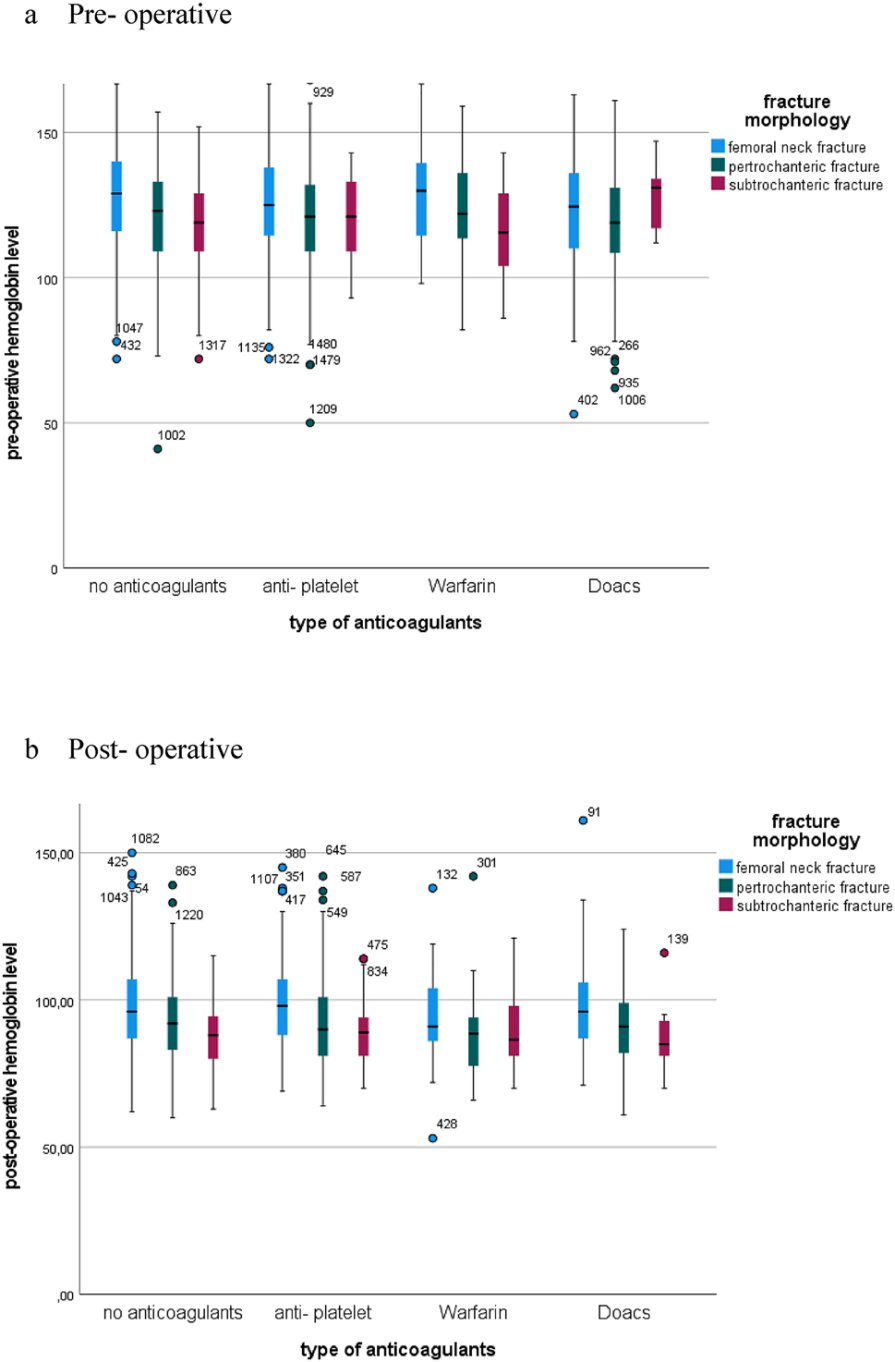
Hemoglobin levels dependent on fracture morphology and anticoagulant therapy

At time of admission after the fracture had occurred average hemoglobin was 12.3 g/l (SD 18.3) and hematocrit 31.4%. Surgery led to a significant drop of hemoglobin levels by 2.9 points (9.4 g/l, SD 14.6), Fig. 2. Postoperative hematocrit was 19.9%, so part of the hemoglobin drop can be attributed to fluid administration. A total of 2,261 transfusions of RBCs were protocolled for 473 patients. On average, if transfusion protocol was required each patient received 4.8 RBCs.

To analyze influencing factors on the total blood loss after surgery of proximal femoral fractures, we performed a multivariate linear regression. Besides fracture morphology, gender, BMI and in-house mortality, the presence of anticoagulants showed a vital influence on the postoperative blood loss. Time to surgery and age did not have any influence as seen in Table 2.

**Table 2:** Influencing factors on blood loss after surgery for proximal femur fracture (linear regression)

Parameter	Reference category	*p* value	Regression coefficient B	95% Confidence interval
Sex	Male	< 0.001	227.803	136,499 to 319,107
Age		0.467	− 1495	− 5527 to 2538
BMI		< 0,001	22,760	13,795 to 31,726
Mortality	No	0.035	258,562	18,517 to 498,608
Fracture type	Femoral neck fracture			
Pertrochanteric fracture		< 0.001	251,067	166,514 to 335,621
Subtrochanteric fracture		< 0.001	606,754	443,035 to 770,472
Anticoagulants	None			
Anti-platelet		0.041	97,852	3792 to 191,912
Warfarin		0.044	180,265	5151 to 355,380
DOAC		0.007	179,816	48,535 to 311,097

**Fig. 3:**
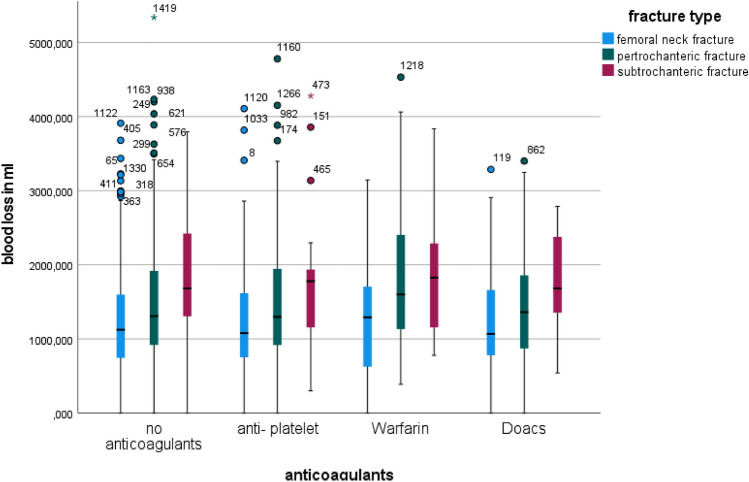
Blood loss dependent on fracture morphology and anticoagulant therapy

Exact calculation of the blood loss showed significant differences between all the fracture types (*p* < 0.001, Kruskal Wallis). On average femoral neck fractures had a blood loss of 1227.5 ml (SD 740.4 ml) whereas pertrochanteric fractures lost 1474.2 ml (SD 830 ml). The highest blood loss could be seen in patients with subtrochanteric femoral fractures with an average loss of 1902.2 ml (SD 1058 ml). Comparing the blood loss depending on anticoagulant therapy patients without anticoagulants and patients on antiplatelet medication showed only a small difference of blood loss (1315.5 ml vs. 1428.8 ml; *p* < 0.041), Fig. 3. There was an increase of blood loss with anticoagulant usage and both Warfarin and DOACs had a significant blood loss (Warfarin: 1620.5 ml, *p* < 0.04; DOACs: 1509.4 ml, *p* < 0.007).

## Discussion

Blood loss after proximal femoral fractures and consecutive surgery is expected and influences the entire postoperative outcome and affects recovery, especially in the geriatric population prone to this type of fracture [9, 10]. Additional medication such as anticoagulants pose a further risk to increased blood loss [17, 18]. Arthroplasty demonstrates a lower amount of blood loss despite being the “more invasive” procedure. This could be due to greater exposure and better possibilities for coagulation due to the open surgical site, whereas intramedullary nailing requires drilling a large trochanteric intramedullary entry point and the inserted nail may not fill out the hole cavity. Bleeders are more difficult to address in minimal invasive procedures.

Studies have shown the actual blood loss to exceed the estimated blood loss [8, 9]. We were able to demonstrate that proximal femoral fractures lead to a large blood loss caused by fracture and surgery. In particular, subtrochanteric fractures lead to significant blood loss. Furthermore, we could demonstrate the additional influence of anticoagulants on blood loss, especially of DOACs and Warfarin.

Similar studies have shown intertrochanteric fractures to have the largest hemoglobin drops postoperatively [19–21]. However, Stacey et al. [19] were not able to show any effect by anticoagulation therapy in a cohort of 119 proximal femoral fractures, possibly due to the small number of patients. Furthermore, age and BMI did not seem to influence his results. The study used the hemoglobin level as an indicator which may be influenced by further factors and may not be quite as reliable as the calculated blood loss.

A study of 546 patients after nailing or screw fixation for proximal femoral fractures showed a comparable blood loss to our results [612 ml (screw), 1812 ml (nail), 1301 (arthroplasty)] [8]. Hidden blood loss was six times higher than seen in surgery and associated with complications and length of hospital stay but not with surgical complications. Patients on antiplatelet therapy had an increased blood loss.

Our standard protocol aims at treating all proximal femur fractures as quickly as possible within 48 h (with the exception of patients on Dabigatran). The treatment algorithm based on renal function and last anticoagulant dose is supported by the literature [22]. We were not able to demonstrate any influence of mean time to surgery. But our data suggest that surgery after 72 h correlates with a higher blood loss, which is supported by Wang et al. [21], who showed a greater drop in hemoglobin levels postoperatively if surgery was carried out more than 48 h after admission. In our case this may well be linked to the presence of anticoagulants. Furthermore, the actual blood loss directly from the fracture will not completely subside before reduction is undertaken and due to the trauma, there can be an important change in hemostasis [20, 21, 23–25]. Mortality did not significantly increase within the first 48 h, which supports our algorithm. Schuetze et al. [26] were even able to demonstrate that early surgery for all patients despite anticoagulants (and reversal by Prothrombin complex) was safe. But Prothrombin complex is associated with thromboembolic events and hip fractures themselves are prone to thromboembolic events, so we believe this to be an unnecessary risk within the first 48 h [27].

Further influential factors on total blood loss have been determined such as general anesthesia (in contrast to spinal anesthesia) as well as reduced bone density, which again is predominantly present in the geriatric population [12, 25]. Positioning of the patient does not seem to have any influence [28]. Intracapsular femoral neck fractures had a smaller hemoglobin drop than extracapsular fractures, which is explained by self-limitation of the initial bleeding by the intact capsule [29].

Hemoglobin levels on admission may also be overestimated by dehydration of patients as well as underestimated by dilution through excessive fluid substitution during surgery and postoperatively. This may lead to incorrect estimation of actual blood loss. Autotransfusion during surgery via cell saver and the administration of tranexamic acid directly to fracture site or intravenously may be a solution to reduce total blood loss and improve recovery and reduce associated complications [30].

There are some limitations to our study. We cannot fully clarify whether the increased blood loss is due to simply delaying surgery as the administration of anticoagulants may influence this result. Furthermore, we did not evaluate tranexamic acid as a potential possibility to reduce blood loss. Factor Xa activity was not recorded as no validated recommendations for undertaking orthopedic surgery are available, but instead we relied on recording the last administration of DOACs and patient specific renal clearance to indicate DOAC activity.

## Conclusions

Hidden blood loss is much greater than expected and estimated, especially for alleged minimally invasive procedures such as intramedullary nailing with trochanteric fractures. Anticoagulants pose an extra risk and a further challenge to surgeons. Besides fracture morphology, gender, and BMI, we were able to demonstrate the vital influence of anticoagulants on total blood loss, especially for DOACs and Warfarin.

It is essential to minimize bleeding intraoperatively and closely monitor postoperative hemoglobin levels with special attention to anticoagulants in the geriatric population as they are vulnerable. Within 48 h there was no increased mortality, so enough time should be given to reduce anticoagulant levels and safely perform surgery. Autotransfusion intraoperatively should be part of the standard treatment protocol for proximal femur fractures to reduce postoperative transfusion rates.
